# 2-Amino-1-methyl-1*H*-imidazol-4(5*H*)-one dimethyl sulfoxide monosolvate

**DOI:** 10.1107/S1600536810038997

**Published:** 2010-10-02

**Authors:** Maya Tutughamiarso, Michael Bolte

**Affiliations:** a Institut für Organische Chemie und Chemische Biologie, Goethe-Universität Frankfurt, Max-von-Laue-Strasse 7, 60438 Frankfurt/Main, Germany; b Institut für Anorganische Chemie, Goethe-Universität Frankfurt, Max-von-Laue-Strasse 7, 60438 Frankfurt/Main, Germany

## Abstract

In the title compound, C_4_H_7_N_3_O·C_2_H_6_OS, creatinine [2-amino-1-methyl-1*H*-imidazol-4(5*H*)one] exists in the amine form. The ring is planar (r.m.s. deviation for all non-H atoms = 0.017 Å). In the crystal, two creatinine mol­ecules form centrosymmetric hydrogen-bonded dimers linked by pairs of N—H⋯N hydrogen bonds. In addition, creatinine is linked to a dimethyl sulfoxide mol­ecule by an N—H⋯O inter­action. The packing shows layers parallel to (120).

## Related literature

For information about creatinine, see: Narayanan & Appleton (1980 ▶). For related structures, see: Bell *et al.* (1995 ▶). For co-crystallization experiments, see: Ton & Bolte (2009 ▶). For hydrogen-bond patterns, see: Bernstein *et al.* (1995 ▶).
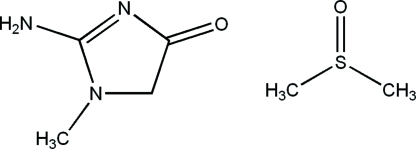


## Experimental

### 

#### Crystal data

C_4_H_7_N_3_O·C_2_H_6_OS
*M* *_r_* = 191.25
Triclinic, 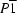

*a* = 5.8997 (10) Å
*b* = 7.3018 (13) Å
*c* = 11.276 (2) Å
α = 75.861 (18)°
β = 83.763 (16)°
γ = 79.694 (13)°
*V* = 462.36 (14) Å^3^
*Z* = 2
Mo *K*α radiation
μ = 0.32 mm^−1^
*T* = 173 K
0.50 × 0.40 × 0.40 mm

#### Data collection

Siemens SMART 1K CCD diffractometer
8161 measured reflections
2997 independent reflections
2597 reflections with *I* > 2σ(*I*)
*R*_int_ = 0.022

#### Refinement

*R*[*F* ^2^ > 2σ(*F* ^2^)] = 0.031
*wR*(*F* ^2^) = 0.082
*S* = 0.98
2997 reflections
120 parameters
H atoms treated by a mixture of independent and constrained refinement
Δρ_max_ = 0.29 e Å^−3^
Δρ_min_ = −0.32 e Å^−3^

### 

Data collection: *SMART* (Siemens, 1995 ▶); cell refinement: *SAINT* (Siemens, 1995 ▶); data reduction: *SAINT*; program(s) used to solve structure: *SHELXS97* (Sheldrick, 2008 ▶); program(s) used to refine structure: *SHELXL97* (Sheldrick, 2008 ▶); molecular graphics: *Mercury* (Macrae *et al.*, 2008 ▶) and *XP* (Sheldrick, 2008 ▶); software used to prepare material for publication: *publCIF* (Westrip, 2010 ▶).

## Supplementary Material

Crystal structure: contains datablocks I, global. DOI: 10.1107/S1600536810038997/bx2310sup1.cif

Structure factors: contains datablocks I. DOI: 10.1107/S1600536810038997/bx2310Isup2.hkl

Additional supplementary materials:  crystallographic information; 3D view; checkCIF report

## Figures and Tables

**Table 1:** Hydrogen-bond geometry (Å, °)

*D*—H⋯*A*	*D*—H	H⋯*A*	*D*⋯*A*	*D*—H⋯*A*
N51—H51*A*⋯O1*D*	0.823 (18)	2.028 (18)	2.8403 (13)	169.3 (17)
N51—H51*B*⋯N1^i^	0.887 (16)	2.040 (16)	2.9225 (14)	172.9 (14)

Symmetry code: (i) 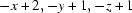.

**Fig. 1.**
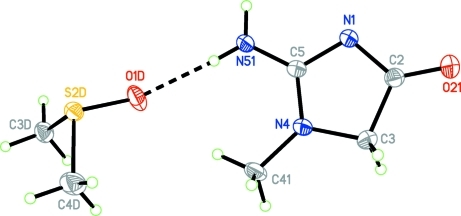
A perspective view of the title compound, showing the atom-numbering scheme. Displacement ellipsoids are drawn at the 50% probability level and H atoms are shown as small spheres of arbitrary radii. The dashed line indicates the N—H···O interaction.

**Fig. 2.**
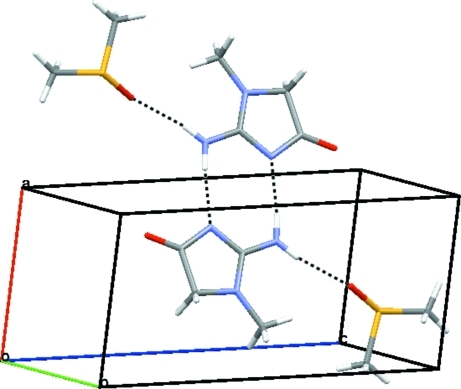
A partial packing diagram of the title compound. Hydrogen bonds are shown as dashed lines.

## supplementary crystallographic information

### Comment

Creatinine is an important end product of nitrogen metabolism and appears in the urine of healthy individuals (Narayanan & Appleton, 1980). Due to the tautomerism, creatinine can exist in two forms, the amine and the imine form. Spectroscopic studies show that the amine form is preferred in the solid state (Bell *et al.*, 1995). To better understand the binding of creatinine to its receptor, we cocrystallized creatinine together with model compounds containing complementary functional groups. During the cocrystallization screening, a creatinine dimethylsulfoxide solvate was obtained. In this structure, the planar creatinine exist also in the amine form (r.m.s. deviation = 0.017 Å for all non-H atoms). The C═N bond is longer than the C—NH_2 _bond [bond lenghts = 1.359 (1) Å and 1.327 (1) Å]. This reversed relation between bond length and nitrogen valence shows that the *π*- electron density is delocalized over the amine-imine group. Creatinine is linked to the solvate molecule by a N—H···O interaction (Fig. 1). This hydrogen-bonded entity is further connected by two N—H···N hydrogen bonds with a *R*^2^_2_(8) pattern forming a centrosymmetric dimer (Bernstein *et al.*, 1995; Fig. 2). The packing shows layers parallel to the (1 2 0) plane.

### Experimental

Single crystals of title compound were obtained by cocrystallization of the commercially available 5-fluorocytosine (2.0 mg) and creatinine (2.1 mg) from dimethylsufoxide (100 µ*L*) at 323 K.

### Refinement

All H atoms were initially located by a difference Fourier synthesis. Subsequently, H atoms bonded to C atoms were refined using a riding model, with methyl C—H = 0.98 Å and secondary C—H = 0.99 Å, and with *U*_iso_(H) = 1.5 *U*_eq_(C) for methyl H or 1.2 *U*_eq_(C) for secondary H. H atoms bonded to N atoms were freely refined.

### Crystal data

**Table:**

C_4_H_7_N_3_O·C_2_H_6_OS	*Z* = 2
*M**_r_* = 191.25	*F*(000) = 204
Triclinic, *P*1	*D*_x_ = 1.374 Mg m^−^^3^
Hall symbol: -P 1	Mo *K*α radiation, λ = 0.71073 Å
*a* = 5.8997 (10) Å	Cell parameters from 131 reflections
*b* = 7.3018 (13) Å	θ = 1.9–32.6°
*c* = 11.276 (2) Å	µ = 0.32 mm^−^^1^
α = 75.861 (18)°	*T* = 173 K
β = 83.763 (16)°	Block, colorless
γ = 79.694 (13)°	0.50 × 0.40 × 0.40 mm
*V* = 462.36 (14) Å^3^	

### Data collection

**Table:**

Siemens SMART 1K CCD diffractometer	2597 reflections with *I* > 2σ(*I*)
Radiation source: normal-focus sealed tube	*R*_int_ = 0.022
graphite	θ_max_ = 32.6°, θ_min_ = 1.9°
ω scans	*h* = −8→8
8161 measured reflections	*k* = −10→10
2997 independent reflections	*l* = −16→16

### Refinement

**Table:**

Refinement on *F*^2^	Primary atom site location: structure-invariant direct methods
Least-squares matrix: full	Secondary atom site location: difference Fourier map
*R*[*F*^2^ > 2σ(*F*^2^)] = 0.031	Hydrogen site location: inferred from neighbouring sites
*wR*(*F*^2^) = 0.082	H atoms treated by a mixture of independent and constrained refinement
*S* = 0.98	*w* = 1/[σ^2^(*F*_o_^2^) + (0.0417*P*)^2^ + 0.166*P*] where *P* = (*F*_o_^2^ + 2*F*_c_^2^)/3
2997 reflections	(Δ/σ)_max_ = 0.001
120 parameters	Δρ_max_ = 0.29 e Å^−^^3^
0 restraints	Δρ_min_ = −0.32 e Å^−^^3^

### Special details

**Table:**

Geometry. All e.s.d.'s (except the e.s.d. in the dihedral angle between two l.s. planes) are estimated using the full covariance matrix. The cell e.s.d.'s are taken into account individually in the estimation of e.s.d.'s in distances, angles and torsion angles; correlations between e.s.d.'s in cell parameters are only used when they are defined by crystal symmetry. An approximate (isotropic) treatment of cell e.s.d.'s is used for estimating e.s.d.'s involving l.s. planes.
Refinement. Refinement of *F*^2^ against ALL reflections. The weighted *R*-factor *wR* and goodness of fit *S* are based on *F*^2^, conventional *R*-factors *R* are based on *F*, with *F* set to zero for negative *F*^2^. The threshold expression of *F*^2^ > σ(*F*^2^) is used only for calculating *R*-factors(gt) *etc*. and is not relevant to the choice of reflections for refinement. *R*-factors based on *F*^2^ are statistically about twice as large as those based on *F*, and *R*- factors based on ALL data will be even larger.

### Fractional atomic coordinates and isotropic or equivalent isotropic displacement parameters (Å^2^)

**Table:**

	*x*	*y*	*z*	*U*_iso_*/*U*_eq_	
N1	0.82954 (15)	0.63634 (13)	0.38132 (8)	0.01974 (17)	
C2	0.72822 (18)	0.72352 (15)	0.27441 (9)	0.01971 (19)	
C3	0.48598 (18)	0.82480 (16)	0.30299 (9)	0.0218 (2)	
H3A	0.4687	0.9638	0.2660	0.026*	
H3B	0.3661	0.7699	0.2743	0.026*	
N4	0.47636 (15)	0.78571 (13)	0.43582 (8)	0.02055 (18)	
C5	0.67429 (17)	0.67487 (14)	0.47372 (9)	0.01701 (18)	
O21	0.81565 (15)	0.72319 (13)	0.17026 (7)	0.02772 (18)	
C41	0.27148 (18)	0.84273 (17)	0.50969 (11)	0.0239 (2)	
H41A	0.1484	0.7740	0.5006	0.036*	
H41B	0.2206	0.9807	0.4824	0.036*	
H41C	0.3068	0.8120	0.5959	0.036*	
N51	0.72130 (16)	0.60912 (14)	0.59037 (8)	0.02144 (18)	
H51A	0.630 (3)	0.632 (2)	0.6476 (16)	0.038 (4)*	
H51B	0.856 (3)	0.534 (2)	0.6059 (14)	0.029 (4)*	
O1D	0.45535 (15)	0.70620 (14)	0.79813 (8)	0.0308 (2)	
S2D	0.29265 (4)	0.65071 (4)	0.90915 (2)	0.02067 (8)	
C3D	0.3041 (2)	0.81135 (17)	1.00396 (10)	0.0247 (2)	
H3D1	0.4563	0.7846	1.0372	0.037*	
H3D2	0.1847	0.7944	1.0715	0.037*	
H3D3	0.2775	0.9433	0.9552	0.037*	
C4D	0.0081 (2)	0.7428 (2)	0.86076 (12)	0.0327 (3)	
H4D1	−0.0012	0.8787	0.8203	0.049*	
H4D2	−0.1033	0.7278	0.9322	0.049*	
H4D3	−0.0275	0.6725	0.8032	0.049*	

### Atomic displacement parameters (Å^2^)

**Table:**

	*U*^11^	*U*^22^	*U*^33^	*U*^12^	*U*^13^	*U*^23^
N1	0.0168 (4)	0.0224 (4)	0.0181 (4)	0.0009 (3)	−0.0003 (3)	−0.0043 (3)
C2	0.0190 (5)	0.0190 (5)	0.0200 (4)	−0.0022 (4)	−0.0009 (3)	−0.0031 (4)
C3	0.0190 (5)	0.0246 (5)	0.0195 (4)	0.0001 (4)	−0.0031 (4)	−0.0025 (4)
N4	0.0145 (4)	0.0256 (4)	0.0195 (4)	0.0024 (3)	−0.0013 (3)	−0.0052 (3)
C5	0.0148 (4)	0.0162 (4)	0.0202 (4)	−0.0018 (3)	−0.0013 (3)	−0.0049 (3)
O21	0.0282 (4)	0.0329 (4)	0.0189 (4)	−0.0009 (3)	0.0024 (3)	−0.0044 (3)
C41	0.0148 (4)	0.0272 (5)	0.0284 (5)	0.0016 (4)	0.0016 (4)	−0.0088 (4)
N51	0.0173 (4)	0.0274 (5)	0.0173 (4)	0.0033 (3)	−0.0008 (3)	−0.0059 (3)
O1D	0.0254 (4)	0.0441 (5)	0.0239 (4)	−0.0052 (4)	0.0076 (3)	−0.0141 (4)
S2D	0.01945 (13)	0.02222 (13)	0.02000 (12)	−0.00115 (9)	−0.00068 (9)	−0.00608 (9)
C3D	0.0250 (5)	0.0292 (5)	0.0210 (5)	−0.0033 (4)	0.0003 (4)	−0.0096 (4)
C4D	0.0202 (5)	0.0450 (7)	0.0337 (6)	−0.0024 (5)	−0.0056 (4)	−0.0110 (5)

### Geometric parameters (Å, °)

**Table:**

N1—C5	1.3591 (13)	C41—H41C	0.9800
N1—C2	1.3644 (13)	N51—H51A	0.823 (18)
C2—O21	1.2306 (13)	N51—H51B	0.887 (16)
C2—C3	1.5269 (15)	O1D—S2D	1.5109 (9)
C3—N4	1.4514 (14)	S2D—C3D	1.7833 (12)
C3—H3A	0.9900	S2D—C4D	1.7844 (13)
C3—H3B	0.9900	C3D—H3D1	0.9800
N4—C5	1.3426 (13)	C3D—H3D2	0.9800
N4—C41	1.4487 (13)	C3D—H3D3	0.9800
C5—N51	1.3269 (13)	C4D—H4D1	0.9800
C41—H41A	0.9800	C4D—H4D2	0.9800
C41—H41B	0.9800	C4D—H4D3	0.9800
			
C5—N1—C2	106.69 (8)	H41A—C41—H41C	109.5
O21—C2—N1	126.20 (10)	H41B—C41—H41C	109.5
O21—C2—C3	124.36 (10)	C5—N51—H51A	123.0 (12)
N1—C2—C3	109.44 (9)	C5—N51—H51B	117.3 (10)
N4—C3—C2	101.23 (8)	H51A—N51—H51B	119.7 (16)
N4—C3—H3A	111.5	O1D—S2D—C3D	106.00 (6)
C2—C3—H3A	111.5	O1D—S2D—C4D	106.16 (6)
N4—C3—H3B	111.5	C3D—S2D—C4D	97.79 (6)
C2—C3—H3B	111.5	S2D—C3D—H3D1	109.5
H3A—C3—H3B	109.3	S2D—C3D—H3D2	109.5
C5—N4—C41	128.18 (9)	H3D1—C3D—H3D2	109.5
C5—N4—C3	108.39 (8)	S2D—C3D—H3D3	109.5
C41—N4—C3	123.06 (9)	H3D1—C3D—H3D3	109.5
N51—C5—N4	124.29 (10)	H3D2—C3D—H3D3	109.5
N51—C5—N1	121.51 (9)	S2D—C4D—H4D1	109.5
N4—C5—N1	114.19 (9)	S2D—C4D—H4D2	109.5
N4—C41—H41A	109.5	H4D1—C4D—H4D2	109.5
N4—C41—H41B	109.5	S2D—C4D—H4D3	109.5
H41A—C41—H41B	109.5	H4D1—C4D—H4D3	109.5
N4—C41—H41C	109.5	H4D2—C4D—H4D3	109.5
			
C5—N1—C2—O21	179.20 (11)	C41—N4—C5—N51	−5.49 (18)
C5—N1—C2—C3	−0.46 (12)	C3—N4—C5—N51	−178.65 (10)
O21—C2—C3—N4	−177.89 (11)	C41—N4—C5—N1	175.64 (10)
N1—C2—C3—N4	1.78 (11)	C3—N4—C5—N1	2.48 (13)
C2—C3—N4—C5	−2.45 (11)	C2—N1—C5—N51	179.85 (10)
C2—C3—N4—C41	−176.03 (10)	C2—N1—C5—N4	−1.25 (12)

### Hydrogen-bond geometry (Å, °)

**Table:**

*D*—H···*A*	*D*—H	H···*A*	*D*···*A*	*D*—H···*A*
N51—H51A···O1D	0.823 (18)	2.028 (18)	2.8403 (13)	169.3 (17)
N51—H51B···N1^i^	0.887 (16)	2.040 (16)	2.9225 (14)	172.9 (14)

Symmetry codes: (i) −*x*+2, −*y*+1, −*z*+1.
